# Proteomics profiling reveals novel proteins and functions of the plant stigma exudate

**DOI:** 10.1093/jxb/ert345

**Published:** 2013-10-22

**Authors:** Juan David Rejón, François Delalande, Christine Schaeffer-Reiss, Christine Carapito, Krzysztof Zienkiewicz, Juan de Dios Alché, María Isabel Rodríguez-García, Alain Van Dorsselaer, Antonio Jesús Castro

**Affiliations:** ^1^Departamento de Bioquímica, Biología Celular y Molecular de Plantas, Estación Experimental del Zaidín (C.S.I.C.), C/ Profesor Albareda 1,18008 Granada, Spain; ^2^Laboratoire de Spectrométrie de Masse Bio-Organique, IPHC-DSA, UdS, CNRS, UMR 7178, 25 rue Becquerel, 67087 Strasbourg, France; ^3^Department of Cell Biology, Institute of General and Molecular Biology, Nicolaus Copernicus University, Gargarina 9, 87–100 Toruń, Poland

**Keywords:** Eastern lily, exudate, olive, proteomics, stigma, secretome.

## Abstract

Proteomic analysis of the stigmatic exudate of *Lilium longiflorum* and *Olea europaea* led to the identification of 51 and 57 proteins, respectively, most of which are described for the first time in this secreted fluid. These results indicate that the stigmatic exudate is an extracellular environment metabolically active, participating in at least 80 different biological processes and 97 molecular functions. The stigma exudate showed a markedly catabolic profile and appeared to possess the enzyme machinery necessary to degrade large polysaccharides and lipids secreted by papillae to smaller units, allowing their incorporation into the pollen tube during pollination. It may also regulate pollen-tube growth in the pistil through the selective degradation of tube-wall components. Furthermore, some secreted proteins were involved in pollen-tube adhesion and orientation, as well as in programmed cell death of the papillae cells in response to either compatible pollination or incompatible pollen rejection. Finally, the results also revealed a putative cross-talk between genetic programmes regulating stress/defence and pollination responses in the stigma.

## Introduction

In angiosperms, pollination begins when pollen comes into contact with the stigma surface and hydrates. Pollen behaviour on the stigma differs among species. In plants with a wet stigma, the surface cells release a viscous secretion in which pollen grains are embedded without any species selectivity (Sanchez *et al.*, 2004). Pollen capture and adhesion is mediated mainly by the stickiness and surface tension of the stigma exudate. This secretion also provides a water source for rapid hydration of pollen and a protective environment for fragile pollen tubes during the first steps of germination. Pollen grains and pollen tubes also uptake nutrients and other compounds from this fluid such as pectic wall precursors (Labarca and Loewus, 1972).

The stigmatic exudate (SE) is synthesized and secreted by differentiated and specialized cells of the stigma surface, which usually protrude as papillae. A Cys-rich protein called stigma-specific protein 1 (STIG1) has been proposed to play a function in the temporal regulation of SE deposition on the stigma surface in tobacco and petunia (Verhoeven *et al.*, 2005). The SE mainly consists of water, polysaccharides, lipids, and proteins (Labarca *et al.*, 1970; Suárez *et al.*, 2012). It has been shown that the tobacco SE has cell-wall-loosening activity, which relies on the action of a lipid transfer protein (LTP) (Nieuwland *et al.*, 2005). Another LTP, named stigma/stylar cysteine-rich adhesin (SCA), is involved in pollen-tube adhesion in lily (Park *et al.*, 2000). In combination with a small stigma protein called chemocyanin, SCA protein also induces chemotropic activity (Kim *et al.*, 2003). Pathogenesis-related proteins (Wakelin and Leung, 2009) and proteinase inhibitors (Busot *et al.*, 2008) also accumulate in the SE, and a function in plant reproduction cannot be ruled out. Despite all these data, the qualitative composition of the SE protein pool and its involvement in pollen–stigma interaction is poorly known to date. In this work, we have carried out the first large-scale analysis of the SE proteins of two species using a proteomic approach in order to find new proteins and provide novel data about the biological and molecular processes that occur in this extracellular fluid. As result, we have provided a comprehensive list of the proteins, and we discuss their physiological relevance in the framework of pollination.

## Materials and methods

### Plant material and sampling

Flowering cuttings of Eastern lily (*Lilium longiflorum* Thunb. cv. ‘White Heaven’) were purchased at a local market. Cuttings were kept in a preservative solution of VIVEFLOR^®^ Complet (Agrosport SL, Barcelona, Spain) and flower buds were left to open at 25 °C, 60% humidity, and a 12h photoperiod under natural irradiation. Flowers were emasculated just before anthesis to keep pollen away from the stigma surface. The stigmatic secretion was collected from flowers by pipetting the drops formed on the stigma surface at 1 day intervals during the 7 days following the anthesis. Three independent samples (100 flowers each) were collected and stored at –20 °C until use. Orange [*Citrus*×*sinensis* (L.) Osbeck], pomegranate (*Punica granatum* L.), and olive (*Olea europaea* L. cv. ‘Picual’) flowers at anthesis were collected from individual trees at the germplasm collection of the Estación Experimental del Zaidín in Granada (Spain). Early flowering branches were put inside paper bags to avoid pollen contamination, cut just before anthesis, and left to open on the bench in the laboratory. Pistils were dissected using a stereomicroscope (M165FC; Leica, Germany) and pictures were taken with a high-resolution digital camera. For each species, three independent samples of SE were collected from 250 pistils each using a very fine synthetic hair paintbrush soaked in 0.1M phosphate buffer (pH 7.0) containing 0.1% (v/v) Tween 20 and 20mM dithiothreitol and stored at −20 °C until use. After brushing, ten olive stigmas were chosen randomly, and fixed and embedded for transmission electron microscopy (TEM) as described below. We found that papillae cells remained intact after exudate collection and there were no changes at the ultrastructural level compared with untreated stigmas (Supplementary Fig. S1 at *JXB* online). The protein content of each sample was measured with a 2-D Quant kit (Amersham Biosciences, Piscataway, NJ, USA) following the manufacturer’s instructions.

### SDS-PAGE

Exudate proteins were precipitated in 9 vols of 20% (w/v) trichloroacetic acid and 0.2% (w/v) dithiothreitol in acetone at −20 °C for 6h. After centrifugation at 10 000*g*, pellets were washed twice in acetone and finally resuspended in 0.5ml of sample buffer [10mM Tris/HCl (pH 8.)0, 1mM EDTA, 5% (v/v) β-mercaptoethanol, 5% (w/v) SDS, 10% (v/v) glycerol and 0.01% (w/v) bromophenol blue]. Exudate protein samples (15 µg of total protein each) were boiled for 5min and then separated by SDS-PAGE according to standard procedures (Laemmli, 1970). Briefly, proteins were separated on 1mm thick gels using a 4% acrylamide stacking gel and a 5–15% acrylamide linear gradient as the resolving gel. Gels were run for 1h at 5 mA per gel and then overnight at a constant current (7 mA per gel). Gels were fixed for 2h in 50% (v/v) ethanol, 3% (v/v) phosphoric acid, followed by three washes in water for 1min each. Gels were stained for 24h with colloidal Coomassie blue solution [0.12 % (w/v) Coomassie Brilliant Blue G250, 10% (w/v) ammonium sulphate, 10% (v/v) phosphoric acid and 20% (v/v) methanol]. Two gels were run for each biological sample (*n*=6 gels). Protein band intensities were profiled from one-dimensional gels using Quantity One v4.6.2 software (Bio-Rad).

For each species, two gels corresponding to two independent samples were systematically cut into slices, and *in gel* digestion was performed with an automated protein digestion system (MassPREP Station; Waters, Manchester, UK). The gel slices were washed three times in a mixture containing 25mM NH_4_HCO_3_:CH_3_CN (1:1, v/v). The cysteine residues were reduced by 50 μl of 10mM dithiothreitol at 57 °C and alkylated with 50 μl of 55mM iodoacetamide. After dehydration with acetonitrile, proteins were cleaved *in gel* with 40 μl of 12.5ng μl^–1^ of modified porcine trypsin (Promega, Madison, WI, USA) in 25mM NH_4_HCO_3_ at 37 °C for 4h. Tryptic peptides were extracted using 60% (v/v) acetonitrile prepared in 0.5% (v/v) formic acid, followed by a second extraction with 100% acetonitrile before nanoflow liquid chromatography coupled to tandem mass spectrometry (nanoLC-MS/MS) analysis.

### LC-MS/MS and data analysis

NanoLC-MS/MS analyses were performed on a nanoACQUITY UltraPerformance LC^®^ System (UPLC^®^) coupled to a quadrupole time-of-flight mass spectrometer (maXis; Bruker Daltonics, Bremen, Germany) equipped with a nano-electrospray source. The UPLC^®^ was equipped with a Symmetry C18 pre-column (20×0.18mm, 5 µm particle size; Waters, Milford, USA) and an ACQUITY UPLC^®^ BEH130 C18 separation column (75 µm×200mm, 1.7 µm particle size, Waters, Milford, USA). Five microlitres of each sample was loaded. The solvent system consisted of 0.1% (v/v) formic acid in water (solvent A) and 0.1% formic acid in acetonitrile (solvent B). Peptides were trapped for 3min at a flow rate of 5 μl min^–1^ with 99% solvent A and 1% solvent B. Elution was performed at 45 °C at a flow rate of 400 nl min^–1^, using a linear gradient of 1–40% solvent B over 35min. The mass spectrometer was operating in positive mode, with the following settings: source temperature was set to 200 °C, while dry gas flow was at 4 l min^–1^. The nano-electrospray voltage was optimized to −4500V. External mass calibration of the time of flight was achieved before each set of analyses using Tuning Mix (Agilent Technologies, Palo Alto, USA) in the mass range of 322–2722 *m*/*z*. Mass correction was achieved by recalibration of the acquired spectra to the applied lock masses [methylstearate ([M+H]^+^ 299.2945 *m*/*z*) and hexakis(2,2,3,3,-tetrafluoropropoxy) phosphazine ([M+H]^+^ 922.0098 *m*/*z*)].

For tandem MS experiments, the system was operated with automatic switching between MS and MS/MS modes in the range of 50–2200 *m*/*z*. The four most abundant peptides (absolute intensity threshold of 1500), preferably doubly, triply, and quadruply charged ions, were selected from each MS spectrum for further isolation and collision induced dissociation fragmentation using argon as the collision gas. Ions were excluded after the acquisition of one MS/MS spectrum and the exclusion was released after 0.6min. The complete system was fully controlled by Hystar 3.2 (Bruker Daltonics).

Raw data (Supplementary Table S1 at *JXB* online) collected during nanoLC-MS/MS analyses were processed and converted into mgf files with DataAnalysis 4.0 (Bruker Daltonics), and all mgf files for a given gel lane were merged using a tool developed in house (available at https://msda.unistra.fr). MS/MS spectra were smoothed using the Savitzky–Golay algorithm with a smoothing width of 0.2 *m*/*z* in one cycle. A charge deconvolution was applied to the MS full scan and the MS/MS spectra.

### Protein identification

Mass data collected during nanoLC-MS/MS were searched using a local Mascot server (v2.2.0,; MatrixScience, London, UK) against an in-house-generated protein database composed of protein sequences of Viridiplantae (Taxonomy ID 33090) and known contaminant proteins such as human keratins and trypsin, extracted from the NCBInr database (version of 19 March 2013) and combined with reverse sequences for all entries using an in-house database generation toolbox (https://msda.unistra.fr; total 2 692 822 entries). Searches were performed without any molecular weight or isoelectric point restrictions, trypsin was selected as the enzyme, carbamidomethylation of cysteine (+57Da) and oxidation of methionine (+16Da) were set as variable modifications and mass tolerances on precursor and fragment ions of 20 ppm and 0.07Da were used, respectively. Mascot (.dat) results were loaded into the Scaffold software (v2.2.0; Proteome Software Inc., Portland, USA) and filtered to evaluate the false discovery rate (Elias and Gygi, 2007). Protein identification was confirmed when at least two peptides with high-quality MS/MS spectra (less than 10 points below Mascot’s threshold score of identity at a 95% confidence level) were identified. A more stringent filter was applied for single peptide identifications, the score of the unique peptide had to be higher than 20 points above the Mascot’s threshold score of identity at the 95% confidence level. These thresholds led to protein identification with a false discovery rate of less than 1.5%.

### Bioinformatic analysis

Unknown protein sequences were analysed to find conserved domains/regions/ motifs using Pfam (http://pfam.sanger.ac.uk) and CDD (http://www.ncbi.nlm.nih.gov/Structure/cdd/wrpsb.cgi) databases. These sequences were also blasted using the DELTA-BLAST algorithm (http://blast.ncbi.nlm.nih.gov/) to search for homology with homologous proteins present in the databases. The SignalP algorithm was used to search for putative signal peptides (http://www.cbs.dtu.dk/services/SignalP/). Functional categorization of proteins was carried out according to both the Fun-CatDB functional catalogue (http://mips.helmholtz-muenchen.de/proj/funcatDB) and the Gene Ontology database (http://www.ebi.ac.uk/QuickGO).

### Electron microscopy

For TEM analysis, unpollinated olive pistils were processed as described previously (Suárez *et al.*, 2012). For scanning electron microscopy (SEM) analysis, unpollinated olive pistils were fixed as described above and post-fixed in 1% (w/v) osmium tetroxide in 100mM sodium cacodylate buffer (pH 7.2) for 2h at 4 °C and dehydrated in a graded ethanol series. The samples were then subjected to critical point drying in a CPD-7501 Drier (Polaron Equipment Ltd, UK), mounted on SEM stubs with double-sided tape, and coated with carbon and gold in an E5000 Sputter Coater (Polaron Equipment Ltd). Samples were observed and photographed in an FEI Quanta 400 FEG ESEM/EDAX Pegasus X4 microscope (Zeiss, Germany), operating at 15kV.

## Results and discussion

### Proteome wide-analysis reveals novel proteins and functions of the plant SE

**Fig. 1.**
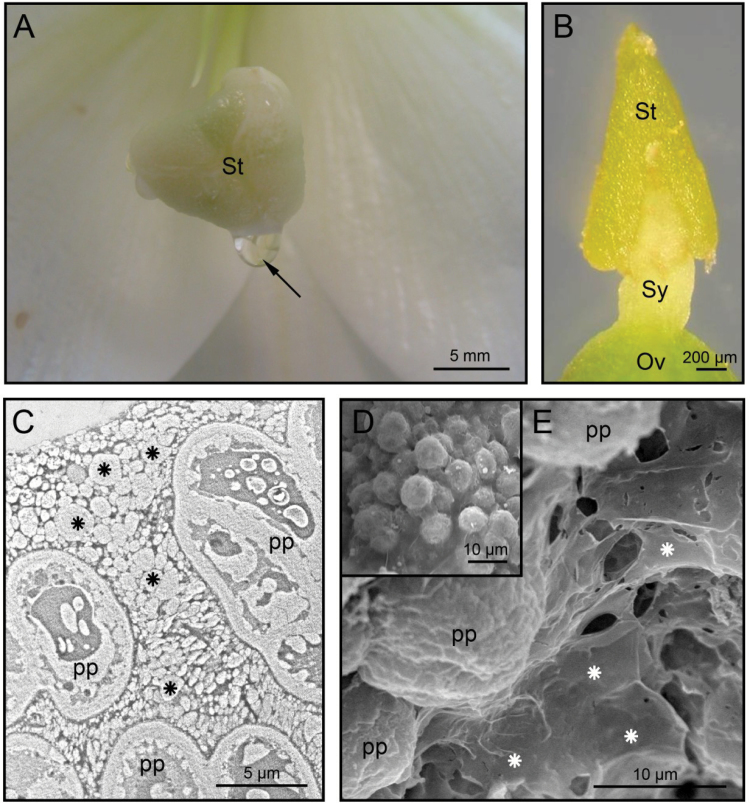
The lily and olive SE. (A) Macroscopic drops of exudate (arrow) formed on the lily stigma surface. (B) Morphology of the olive stigma. (C) TEM photomicrograph showing the olive SE (asterisks) at anthesis. (D, E) SEM photomicrographs of the olive SE (asterisks) as above. Ov, ovary; pp, papillae; St, stigma; Sy, style. (This figure is available in colour at *JXB* online.)

**Fig. 2.**
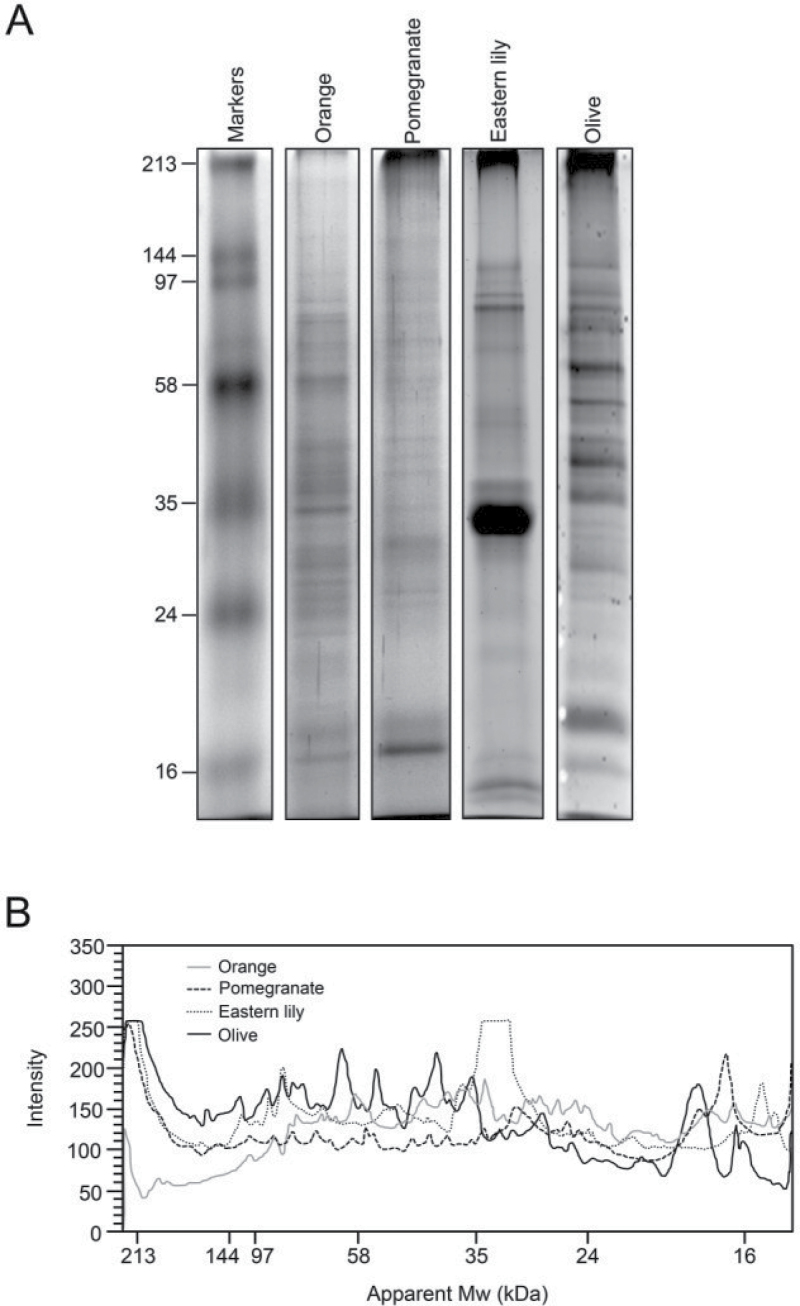
Protein profile of the SE in several plant species. (A) Exudate protein samples (~15 µg per lane) were separated by SDS-PAGE and stained with Coomassie Brilliant Blue. Protein markers are displayed on the left. (B) The gel band intensities were profiled for each species.

Lily and olive were chosen for this study on the basis of their divergent taxonomical position, pistil anatomy, exudate composition, geographical distribution, cultivation purposes, and sampling procedure (Table 1). In lily, the SE was deposited as macroscopic drops on the stigma surface (Fig. 1A). In the olive, this secretion appeared as a thin layer of vesicular aspect that coated the stigma surface (Fig. 1B), which was only visible under the electron microscope (Fig. 1C–E). The protein profiles of the SE in several angiosperms showed quantitative and qualitative differences (Fig. 2). Because Eastern lily and olive genomes are not yet fully annotated, the MS/MS-based identification strategy was not trivial. We evaluated in parallel a classical approach using a database search algorithm (Mascot) and a *de novo* sequencing approach, best adapted for organisms not well represented in available protein databases. The latter strategy implies the individual treatment of the peptide fragmentation spectra to deduce a complete or partial peptide sequence tag and a subsequent sequence homology search using BLAST alignments (Castro *et al.* 2005; Catusse *et al.* 2008). The results of the two approaches were compared and the *de novo* sequencing approach did not allow identification of additional proteins when compared with classical database searches. It only allowed the identification of a few additional peptides and minor increases in sequence coverage of several proteins already identified using database search algorithms. Additionally, the statistical methods used to validate identifications by classical database search algorithms (target/decoy approaches) are not applicable when *de novo* sequencing is required. Therefore, merging identification results obtained with those two approaches is a difficult task. Therefore, only classical database search results are presented in this paper.

**Table 1.** Main features of plants chosen for this study

Feature	Lily	Olive
Taxonomy	Monocots	Dicots
Type of stigma	Wet/Papillary	Wet/Papillary
Exudate composition	Aqueous	Lipidic
Stigma/style anatomy	Hollow	Solid
Pollination	Entomophilous	Anemophilous
Geographical distribution	Japan to Philippines	Mediterranean
Cultivation purpose	Ornamental	Agricultural
Sampling method	Pipetting	Brushing

**Fig. 3.**
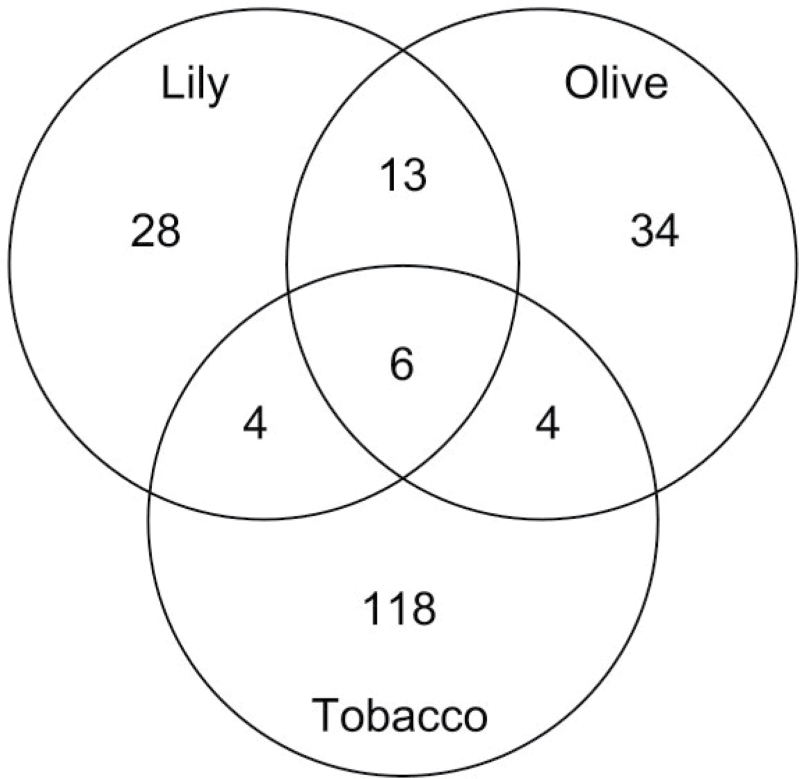
Venn diagram comparing the total number of proteins identified in Eastern lily, tobacco, and olive SEs. Tobacco data from Sang *et al.* (2012).

Our data illustrate the fact that plant proteomes are starting to be very well represented in available protein databases, which makes classical database search algorithms applicable, even for not yet fully sequenced and annotated plant species. Thus, using this approach, we identified 51 different proteins in lily and 57 in olive, of which only 13 were present in both SEs (Supplementary Table S2 at *JXB* online). Lily and olive proteins were classified into 64 and 81 families, respectively, according to the Pfam database (Bateman *et al.*, 2004), of which only 19 were identified in both exudates (Supplementary Table S2). Four proteins identified in lily (chitinase, cysteine proteinase, α-galactosidase, and α-mannosidase), another four in olive (invertase, fructose bi-P aldolase and ATPase α- and β-subunits), and six in both species (malate dehydrogenase, triose-phosphate isomerase, endo-β-1,3-glucanase, Hsp70, LRR receptor kinase and thaumatin-like protein) were recently reported in tobacco stigma surface eluates (Sang *et al.*, 2012) (Fig. 3). Despite our data suggesting functional divergences between lily and olive SEs, a laborious process of experimental validation would be necessary to probe such differences. Instead, proteomics profiling discrepancies might rely on the fact that both species are not fully annotated in databases; hence, some peptides could not be identified. Moreover, sampling methods also differed between the two species studied. Thus, several proteins identified in the olive SE (e.g. apoplastic invertase, accession no. 359431001) might instead be associated with the cell wall/apoplast and have been collected during sampling.

About 70% of lily and 35% of olive proteins (Supplementary Table S2) were predicted to contain a signal peptide (SignalP v4.0 Server; Petersen *et al.*, 2011). Secretion of signal peptide-containing proteins might occur by the canonical endoplasmic reticulum–Golgi exocytic pathway. Indeed, both the lily and the olive stigmatic papillae possess the ultrastructural features typical of secretory cells (Miki-Hiroshige *et al.*, 1987; Suárez *et al.*, 2012). However, about 30% of matched proteins in Eastern lily and up to 65% in olive lacked a signal peptide. Similarly, about 45 and 80% of proteins present in *Arabidopsis* cell culture and pea root cap secretomes, respectively, lack an identifiable signal sequence (Oh *et al.*, 2005; Wen *et al.*, 2007). Most of the SE proteins were actually identified in other plant species. Moreover, signal peptide-predicting algorithms are subject to failure. Therefore, contrary to predictions, these lily and olive proteins may indeed contain signal sequences.

**Fig. 4.**
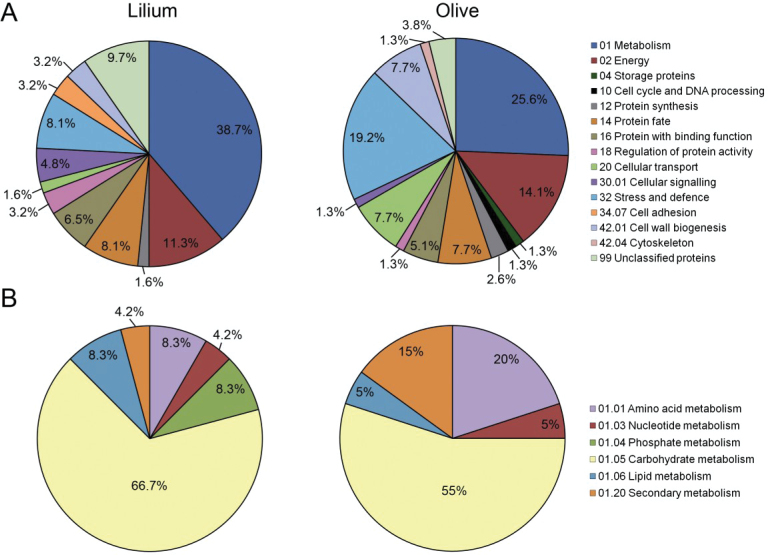
Distribution of functional classes of lily and olive stigma exudate proteins. (A) Pie chart of main functional categories (Ruepp *et al.*, 2004). (B) Pie chart of secondary functional categories referred to metabolism (Ruepp *et al.*, 2004). The percentage of proteins in each category is shown.

Based on ontological classifications (Ruepp *et al.*, 2004; The Gene Ontology Consortium, 2010), we found that the SE might be involved in at least 80 different biological processes and 97 molecular functions (Supplementary Table S2). Major functional categories included one-carbon and carbohydrate metabolism, protein fate, cell-wall organization, cell signalling, and response to biotic and abiotic stresses (Fig. 4). Below, we discuss the most outstanding findings revealed by the present study in the context of sexual reproduction in angiosperms.

### The SE proteome shows a markedly catabolic profile

*O*-Glycosyl hydrolases (GHs) were by far the most abundant group of proteins in the lily SE. This indicated the importance of polysaccharide catabolism. Indeed, arabinogalactan proteins (AGPs) and pectins are the major components of lily SE (Labarca *et al.*, 1970). In a pioneer study, Labarca and Loewus (1972) demonstrated that lily pollen tubes uptake carbohydrate wall precursors from the SE. In this context, a plausible scenario would be one in which some GH enzymes such as β-galactosidases, β-xylosidases, and α-arabinofuranosidases would contribute to degrade large polysaccharides secreted by papillae to smaller units, allowing their incorporation into the pollen tube. We also identified an endo-β-mannosidase, which hydrolyses the Manβ1-4GlcNAc linkage in the trimannosyl core structure of *N*-glycans (Ishimizu *et al.*, 2004), as well as a putative α-mannosidase involved in the hydrolysis of Manα1-3Man and Manα1-6Man linkages (Hossain *et al.*, 2009). Based on our findings, it is tempting to suggest that these two mannosidases might cooperate to degrade high-mannose-type *N*-glycans to GlcNAcβ1-4GlcNAc on glycoproteins secreted by the stigma. For instance, *N,N′*-diacetyl chitobiose (GlcNAcβ1-4GlcNAc) is a major *N*-glycan of S-RNase, a glycoprotein associated with gametophytic self-incompatibility (Woodward *et al.*, 1992).

A second group of hydrolases in the lily SE consisted of two classes of proteolytic enzymes, namely cysteine and aspartic proteases. Proteases are normally found at different intracellular locations, but various ones accumulate extracellularly in the transmitting tissue (Vieira *et al.*, 2001). Accordingly, they might participate in limited proteolysis reactions important for pollen–pistil interaction events including pollen adhesion and recognition. Alternatively, these proteases might generate or modify precursors or signals for directional tube growth.

Finally, a third group of hydrolases included GDSL-like lipases, which were identified in the SE of both species. Extracellular GDSL lipases have been identified in several plant secretions including nectar (Kram *et al.*, 2008). Pollen coat lipids modulate water transfer to desiccated pollen in dry-type stigmas (Wolters-Arts *et al.*, 2002). The lipid-rich exudate of wet stigmas is thought to be functionally analogous to the pollen coat. Interestingly, a pollen coat GDSL-like lipase is necessary for efficient hydration of *Arabidopsis* pollen (Updegraff *et al.*, 2009). In this context, GDSL-like lipases might promote pollen hydration by modifying lipid composition at the pollen–SE interface. As suggested previously for nectar GDSL-like lipases, it is also plausible that these enzymes might function in preventing microbial growth (Kram *et al.*, 2008).

### Is there cross-talk between genetic programmes regulating stress/defence and pollination responses in the stigma?

The sugar-enriched SE is particularly susceptible to infection by fungi and bacteria. This fact may explain the presence of several pathogenesis-related protein families and other defence-related proteins. However, these defence-related genes might also function in response to pollination. For instance, chitinases might be involved in the degradation of AGPs, and the released carbohydrates might function as signalling molecules (Van Hengel *et al.*, 2002). Moreover, the stigmas of healthy mature petunia flowers show β-1,3-glucanase activity, but this enzyme has no antifungal activity (Wakelin and Leung, 2009). Papain (C1)- and subtilisin-like proteases are also involved in local and systemic defence responses, including that against incompatible pollen (van der Hoorn, 2008). The pollen of lily and other plants releases cyclophilins to the extracellular medium under unfavourable conditions (Yokota *et al.*, 2004), and pollen germination is inhibited in the medium into which cyclophilins are released. Some other identified proteins including lectins, protein inhibitors of cell-wall degrading enzymes [e.g. polygalacturonase-inhibiting proteins (PGIPs) and pectin methylesterases (PME) inhibitors (PMEIs)], and reactive oxygen species detoxifying enzymes might also function in response to pollination (Wilkins *et al.*, 2011).

Interestingly, several aspects of sexual reproduction in the olive, such as stigmatic receptivity, pollen performance, and self-incompatibility relationships, are notably affected by temperature (Koubouris *et al.*, 2009). In response to heat stress, plants synthesize a group of proteins called heat-shock proteins (HSPs), which act as molecular chaperones for other proteins. Interestingly, the olive stigma secretes HSP60, HSP70, and HSP90 proteins. Extracellular HSPs are emerging as important mediators in cell signalling in response to stress (Calderwood *et al.*, 2007). These proteins might also modulate temperature-dependent reproductive processes in the olive.

### The SE may regulate pollen-tube growth by selective degradation of cell-wall polysaccharides

Several enzymes secreted into the SE might regulate pollen tube growth in the stigma through selective degradation of tube wall pectins (Table 2). Polygalacturonases hydrolyse homogalacturonans to oligogalacturonides, which can act as signalling molecules (Ridley *et al.*, 2001). The exogenous addition of polygalacturonase to the culture medium promotes early pollen-tube wall extension, probably as result of an increase in plasticity in the tip region (Roggen and Stanley, 1969). PMEs found in the SE of olive and lily contain N-terminal pro-regions with homology to PMEIs. Interestingly, a recent study suggested that PMEIs might be internalized by endocytosis at the flanks of the pollen tube tip, regulating pollen-tube wall stability by locally inhibiting pollen PME activity (Röckel *et al.*, 2008). According to our findings, it is tempting to suggest that these PMEI isoforms might be of stigmatic origin. A PGIP was identified in the olive SE, but whether this protein regulates polygalacturonase activity in the stigma is unknown. Finally, a pectin acetylesterase was also identified in the olive SE. This enzyme has been suggested to function as an important structural regulator by modulating the status of pectin acetylation, thereby affecting cell extensibility (Gou *et al.*, 2012). In addition to pectinases, bifunctional α-arabinosidases/β-xylosidases and β-galactosidases might be also involved in degradation of arabinan and galactan side chains of pectins, respectively.

**Table 2.** List of cell-wall-modifying enzymes identified in the stigma exudates of lily and olive

Olive^*a*^	Lily^*a*^	Cell-wall process
Dhurrinase (*Sorghum bicolor*) [1127575]	–	Cellulose, xyloglucan catabolism
β-Glucosidase (*Glycine max*) [356565758]	β-glucosidase (*Picea sitchensis*) [116786797]	Cellulose, xyloglucan catabolism
–	β-Xylosidase/α-arabinofuranosidase (*Medicago sativa*) [292630922]	Arabinan catabolism
–	Exo-1,3-β-glucanase (*Lilium longiflorum*) [46091271]	1,3-β-glucan (callose) catabolism
UDP-glucose 6-dehydrogenase (*Arabidopsis lyrata*) [297805822]	–	Unknown
Xyloglucan endotransglucosylase (*Actinidia deliciosa*) [187372962]	–	Xyloglucan catabolism
Endo-1,3-β-glucanase (*Olea europaea*) [75165700]	Endo-1,3-β-glucanase (*Zea mays*) [223945355]	1,3-β-glucan (callose) catabolism
–	α-Galactosidase (*Selaginella moellendorffii*) [302753418]	Galactomannan catabolism
Polygalacturonase (*Ricinus communis*) [255553564]	Polygalacturonase (*Vitis vinifera*) [359480238]	Homogalacturonan catabolism
–	β-Galactosidase, GH family 35 (*Sorghum bicolor*) [242090613]	Galactan catabolism
–	β-Galactosidase, GH family 42 (*Cucumis sativus*) [449489943]	Galactan catabolism
Pectinesterase (*Olea europaea*) [449061779]	Pectinesterase (*Citrus sinensis*) [2098713]	Homogalacturonan catabolism
Pectin acetylesterase (*Ricinus communis*) [255557763]	–	Homogalacturonan catabolism
Lipid transfer protein (*Olea europaea*) [154257299]	–	Cellulose, xyloglucan catabolism
–	Glycerophosphodiester Pdiesterase (*Ricinus communis*) [255547185]	Unknown
α-1,4-Glucan-protein synthase (*Picea sitchensis*) [116779321]	–	Cellulose biosynthesis

^*a*^The accession no. in Supplementary Table S2 is given in parentheses.

Two classes of enzyme activities associated with callose degradation were present in the SE (Table 2). Thus, endo-1,3-β-glucanases are involved in cleavage of glucan polymers to glucooligosaccharides, which, in turn, are degraded to glucose units by exo-1,3-β-glucanases. Application of endo-1,3-β-glucanase to the culture medium at the beginning of germination increases pollen-tube growth rate, probably as result of callose degradation inside the apertural intine (Roggen and Stanley, 1969). On the other hand, exo-β-1,3-glucanases might be involved in controlled degradation of callose just behind the pollen tube tip to facilitate tip expansion, to prevent the tip from clogging and/or to increase the porosity of the callosic wall for pollen tube–stigma communication (Takeda *et al.*, 2004).

A recent study showed that cellulose has a role in the mechanical stabilization of the pollen tube subapical transition region (Aouar *et al.*, 2010). Addition of cellulase at low concentration to the germination medium has similar effects to pectinase (Roggen and Stanley, 1969). Accordingly, we found putative cellulose degrading β-glucosidases in the SE of both species, as well as a dhurrinase and an LTP in the olive SE (Table 2). Controlled digestion of cellulose might release trapped xyloglucans, resulting in enhanced wall extensibility (Ohmiya *et al.*, 2000). Cellulose microfibrils form a stable three-dimensional network by interacting with hemicelluloses. Therefore, the dynamic nature of this cross-linking might also be a major factor modulating the tube wall expansion rate. The SE also contains a set of enzymes involved in hemicellulose restructuration. Thus, the action of xyloglucan endotransglucosylase and β-glucosidase might release xyloglucan oligosaccharides and cause the cell-wall loosening (Fry *et al.*, 1992). In addition, α-xylosidase and β-galactosidase might cleave xylosylated and galactosylated xyloglucan oligosaccharides. Studies on *Arabidopsis* pollen have shown that xylosidases are involved in cell expansion during pollen germination (Hrubá *et al.*, 2005). Other putative cell-wall-loosening enzymes identified in the SE included several LTPs in olive. These proteins are purportedly involved in pollen-tube growth regulation (Nieuwland *et al.*, 2005). We also identified a glycerophosphoryl diester phosphodiesterase (GDP) in the lily SE. Interestingly, SHV3, a GDP-like protein required for root hair tip growth, was recently described as a novel factor involved in cell-wall organization (Parker *et al.*, 2000).

### Pollen-tube adhesion and guidance is mediated by SE proteins

Cell–cell adhesion is one of the key features for proper pollen germination and pollen-tube growth through the pistil tissues. Several proteins identified in the lily SE are involved in cell–cell adhesion and chemotropic phenomena, including two SCA isoforms, a chemocyanin protein, and a fasciclin-like domain (FAS) protein. The SCA protein is a small cysteine-rich LTP necessary for pollen-tube adhesion to the stylar matrix (Park *et al.*, 2000). Chemocyanin is a small basic protein that induces pollen chemotropism and its activity is potentiated by SCA (Kim *et al.*, 2003). Two LTPs were also identified in the olive SE. The fasciclin-like domain protein identified in lily shows homology with a group of AGPs, called fasciclin-like AGPs (FLAs), which have been shown to function as adhesion molecules in cell walls (Shi *et al.*, 2003). Plant lectins sited at the cell surface or extracellularly are rare, but a function in cell adhesion cannot be ruled out. Finally, polygalacturonases and other cell-wall-remodelling enzymes may also regulate pollen-tube adhesion by selective degradation of cell-wall pectins (Atkinson *et al.*, 2002).

### Cell–cell recognition and signalling in the SE

Some of the proteins identified in the SE are typically involved in cell–cell recognition and signalling. Thus, we identified receptor-like protein kinases in the SE of both lily and olive. Interestingly, the first signal transduction mechanism described in pollen comprised two receptor-like protein kinases (RLKs), namely LePRK1 and LePRK2 (Muschietti *et al.*, 1998). In tomato, the extracellular domain of LePRK2 interacts with a pollen cysteine-rich extracellular protein called LAT52 (Tang *et al.*, 2002). LePRK1 and LePRK2 associate in mature pollen, but dissociate in the presence of the stigmatic factor LeSTIG1, which could displace binding of LAT52, thus triggering an autocrine signalling cascade in the pollen tube (Tang *et al.*, 2004).

The lily SE also contained a phospholipase C (PLC). There is evidence suggesting that PLCs are involved in lipid-mediated modulation of reproductive processes such as pollen-tube growth in petunia (Dowd *et al.*, 2006) and self-incompatibility in poppy (Franklin-Tong *et al.*, 1996). However, no extracellular PLCs have been described in plants to date. Interestingly, *Pseudomonas aeruginosa* secretes an extracellular PLC that is required for phospholipid chemotaxis (Barker *et al.*, 2004).

The olive SE also contained a 14-3-3 protein. These proteins function as cellular integrators of different signalling pathways (DeLille *et al.*, 2001). The presence of secreted 14-3-3 proteins has been reported in the extracellular matrix of the green alga *Chlamydomonas* (Voigt and Frank, 2003) and in pea root tip exudate (Wen *et al.*, 2007). In *Chlamydomonas*, secreted 14-3-3 proteins appear to play a role in cross-linking of hydroxproline-rich glycoproteins in the cell wall (Voigt and Frank, 2003). The presence of a 14-3-3 protein in the olive SE suggests interactions with different targets. The search for protein partners will help to elucidate the function of this protein in the SE. A PGIP was also identified in the olive SE. Because olive pollen secretes a polygalaturonase (unpublished data), its interaction with the stigma PGIP may constitute a recognition signal between the pollen and the stigma in this species.

Regulation of reactive oxygen species is key for plant reproduction (McInnis *et al.*, 2006; Wilkins *et al.*, 2011). Several antioxidant enzymes were identified in the SE, including a Cu/Zn-superoxide dismutase and a putative bifunctional acid phosphatase/peroxidase enzyme in lily, and an annexin in olive. Stigmatic peroxidases might participate indirectly through H_2_O_2_ catabolism in signalling networks that modulate, for instance, species–species pollen recognition (McInnis *et al.*, 2006). Interestingly, some plant annexins exhibit peroxidase activity (Laohavisit *et al.*, 2009). However, other functions cannot be ruled out. For instance, annexins might modulate Ca^2+^ influx, as shown recently in maize (Laohavisit *et al.*, 2009).

### Programmed cell death at the stigma: a putative role for SE proteins

Senescence is the final stage of stigma development in unpollinated pistils (Suárez *et al.*, 2012). Moreover, in pollinated pistils, stigmatic cells undergo programmed cell death after both compatible pollination and incompatible pollen rejection (Wu and Cheung, 2000). The lily SE contained several proteins potentially involved in programmed cell death including several proteases and their inhibitors (Panavas *et al.*, 1999), an S1-type nuclease homologous to SAP6 (senescence-associated protein 6) from *Hemerocallis* (Solomon *et al.*, 1999), and a trehalase (Fernandez *et al.*, 2010).

### Unexpected proteins and functions in the SE

Among the unexpected proteins, an *N*-acetylglucosaminidase involved in the remodelling of *N*-acetylglucosamine (GlcNAc) residues of extracellular proteoglycans and a DUF642 protein were identified in the lily SE. The heparan sulphate polysaccharide has not been found in plants so far, but some AGPs present in the extracellular matrix contain GlcNAc residues (van Hengel *et al.*, 2001). Therefore, it is likely that the substrate of this enzyme possesses signalling/regulatory properties. DUF642 proteins interact with cell-wall polysaccharides *in vitro* (Vázquez-Lobo *et al.*, 2012). We also identified actin, a few enzymes of the methylation cycle, calnexin and calreticulin proteins, and several membrane-bound ATPases in the olive SE. All these proteins were identified previously in the pea root cap secretome (Wen *et al.*, 2007). Actin is associated with plasmodesmata within the cell wall (White *et al.*, 1994). Both SEs also contained translational elongation factors and several intracellular glycolytic enzymes. Interestingly, the plant extracellular adhesion vitronectrin-like 1 protein (PVN1) is nearly identical to the translational elongation factor 1α (Zhu *et al.*, 1994). Moreover, some glycolytic enzymes have been found in apoplastic (Chivasa *et al.*, 2005) and extracellular (Oh *et al.*, 2005; Wen *et al.*, 2007) fluids, but their biological relevance is unknown.

## Supplementary data

Supplementary data is available at *JXB* online.

Supplementary Fig. S1. (A) Transmission electron microscopy (TEM) photomicrograph of an olive stigma before pollination showing papillae ultrastructure and the presence of a copious exudate covering its surface. (B) TEM photomicrograph as above showing intact papillae after exudate collection by brushing.

Supplementary Table S1. Raw data files from nanoLC-MS/MS analysis of Eastern lily and olive stigma exudates.

Supplementary Table S2. List of *Lilium* and olive stigmatic exudate proteins identified by nanoLC-MS/MS analysis.

Supplementary Data

## Abbreviations:

AGP: arabinogalactan protein
GH: *O*-glycosyl hydrolase
HSP: heat-shock protein
LTP: lipid transfer protein
nanoLC-MS/MS: nanoflow liquid chromatography coupled to tandem mass spectrometry
PLC: phospholipase C
PME: pectin methylesterase
PMEI: PME inhibitor
RLK: receptor-like protein kinase
SE: stigmatic exudate
SEM: scanning electron microscopy
TEM: transmission electron microscopy.

## References

[CIT0001] Aouar L Chebli Y Geitmann A 2010 Morphogenesis of complex plant cell shapes: the mechanical role of crystalline cellulose in growing pollen tubes. Sexual Plant Reproduction 23, 15–27 20165960 10.1007/s00497-009-0110-7

[CIT0002] Atkinson RG Schröder R Hallett IC Cohen D MacRae EA 2002 Overexpression of polygalacturonase in transgenic apple trees leads to a range of novel phenotypes involving changes in cell adhesion. Plant Physiology 129, 122–133 12011344 10.1104/pp.010986 PMC155877

[CIT0003] Barker AP Vasil AI Filloux A Ball G Wilderman PJ Vasil ML 2004 A novel extracellular phospholipase C of *Pseudomonas aeruginosa* is required for phospholipid chemotaxis. Molecular Microbiology 53, 1089–1098 15306013 10.1111/j.1365-2958.2004.04189.x

[CIT0004] Bateman A Coin L Durbin R 2004 The Pfam protein families database. Nucleic Acids Research 32, D138–D141 14681378 10.1093/nar/gkh121 PMC308855

[CIT0005] Busot GY McClure B Ibarra-Sánchez CP Jiménez-Durán K Vázquez-Santana S Cruz-García F 2008 Pollination in *Nicotiana alata* stimulates synthesis and transfer to the stigmatic surface of NaStEP, a vacuolar Kunitz proteinase inhibitor homologue. Journal of Experimental Botany 59, 3187–3201 18689443 10.1093/jxb/ern175 PMC2504342

[CIT0006] Calderwood SK Mambula SS Gray PJ Jr 2007 Extracellular heat shock proteins in cell signaling and immunity. Annals of the New York Academy of Sciences 1113, 28–39 17978280 10.1196/annals.1391.019

[CIT0007] Castro AJ Carapito C Zorn N Magné C Leize E Van Dorsselaer A Clément C 2005 Proteomic analysis of grapevine (*Vitis vinifera* L.) tissues subjected to herbicide stress. Journal of Experimental Botany 56, 2783–2795 16216849 10.1093/jxb/eri271

[CIT0008] Catusse J Strub JM Job C Van Dorsselaer A Job D 2008 Proteome-wide characterization of sugarbeet seed vigor and its tissue specific expression. Proceedings of the Natural Academy of Sciences, USA 105, 10262–10267 10.1073/pnas.0800585105 PMC2474813 18635686

[CIT0009] Chivasa S Ndimba BK Simon WJ Lindsey K Slabas AR 2005 Extracellular ATP functions as an endogenous external metabolite regulating plant cell viability. Plant Cell 17, 3019–3034 16199612 10.1105/tpc.105.036806 PMC1276027

[CIT0010] DeLille J Sehnke PC Ferl RJ 2001 The *Arabidopsis thaliana* 14-3-3 family of signaling regulators. Plant Physiology 126, 35–38 11351068 10.1104/pp.126.1.35 PMC1540106

[CIT0011] Dowd PE Coursol S Skirpan AL Kao T-H Gilroy S 2006 Petunia phospholipase C1 is involved in pollen tube growth. Plant Cell 18, 1438–1453 16648366 10.1105/tpc.106.041582 PMC1475500

[CIT0012] Elias JE Gygi SP 2007 Target-decoy search strategy for increased confidence in large-scale protein identifications by mass spectrometry. Nature Methods 4, 207–214 17327847 10.1038/nmeth1019

[CIT0013] Fernandez O Béthencourt L Quero A Sangwan RS Clément C 2010 Trehalose and plant stress responses: friend or foe? Trends in Plant Science 15, 409–417 20494608 10.1016/j.tplants.2010.04.004

[CIT0014] Franklin-Tong VE Drobak BK Allan AC Watkins P Trewavas AJ 1996 Growth of pollen tubes of *Papaver rhoeas* is regulated by a slow-moving calcium wave propagated by inositol 1,4,5-trisphosphate. Plant Cell 8, 1305–1321 12239415 10.1105/tpc.8.8.1305 PMC161246

[CIT0015] Fry SC Smith RC Renwick KF Martin DJ Hodge SK Matthews KJ 1992 Xyloglucan endotransglycosylase, a new wall-loosening enzyme activity from plants. Biochemical Journal 282, 821–828 1554366 10.1042/bj2820821 PMC1130861

[CIT0016] Gou JY 2012 Acetylesterase-mediated deacetylation of pectin impairs cell elongation, pollen germination, and plant reproduction. Plant Cell 24, 50–65 22247250 10.1105/tpc.111.092411 PMC3289554

[CIT0017] Hossain MA Nakamura K Kimura Y 2009 α-mannosidase involved in turnover of plant complex type N-glycans in tomato (*Lycopersicum esculentum*) fruits. Biosciences, Biotechnology and Biochemistry 73, 140–146 10.1271/bbb.80561 19129634

[CIT0018] Hrubá P Honys D Twell D Capková V Tupý J 2005 Expression of β-galactosidase and β-xylosidase genes during microspore and pollen development. Planta 220, 931–940 15517348 10.1007/s00425-004-1409-0

[CIT0019] Ishimizu T Sasaki A Okutani S Maeda M Yamagishi M Hase S 2004 Endo-β-mannosidase, a plant enzyme acting on *N*-glycan: purification, molecular cloning, and characterization. Journal of Biological Chemistry 279, 38555–38562 15247239 10.1074/jbc.M406886200

[CIT0020] Kim ST Mollet J-C Dong J Zhang K Park S-Y Lord EM 2003 Chemocyanin, a small basic protein from the lily stigma, induces pollen tube chemotropism. Proceedings of the Natural Academy of Sciences, USA 100, 16125–16130 10.1073/pnas.2533800100 PMC307703 14671326

[CIT0021] Koubouris GC Metzidakis IT Vasilakakisb MD 2009 Impact of temperature on olive (*Olea europaea* L.) pollen performance in relation to relative humidity and genotype. Environmental and Experimental Botany 67, 209–214

[CIT0022] Kram BW Bainbridge EA Perera MA Carter C 2008 Identification, cloning and characterization of a GDSL lipase secreted into the nectar of *Jacaranda mimosifolia* . Plant Molecular Biology 68, 173–183 18553138 10.1007/s11103-008-9361-1

[CIT0023] Labarca C Kroh M Loewus F 1970 The composition of stigmatic exudate from *Lilium longiflorum*. Labelling studies with myo-inositol, d-glucose and l-proline. Plant Physiology 46, 150–156 16657408 10.1104/pp.46.1.150 PMC396550

[CIT0024] Labarca C Loewus F 1972 The nutritional role of pistil exudate in pollen tube wall formation in *Lilium longiflorum*: utilization of injected stigmatic exudate. Plant Physiology 50, 7–14 16658136 10.1104/pp.50.1.7 PMC367306

[CIT0025] Laemmli UK 1970 Cleavage of structural proteins during the assembly of the head of bacteriophage T4. Nature 227, 680–685 5432063 10.1038/227680a0

[CIT0026] Laohavisit A Mortimer JC Demidchik V 2009 *Zea mays* annexins modulate cytosolic free Ca^2+^, form a Ca^2+^-permeable conductance and have peroxidase activity. Plant Cell 21, 479–493 19234085 10.1105/tpc.108.059550 PMC2660635

[CIT0027] McInnis SM Desikan R Hancock JT Hiscock SJ 2006 Production of reactive oxygen species and reactive nitrogen species by angiosperm stigmas and pollen: potential signalling crosstalk? New Phytologist 172, 221–228 16995910 10.1111/j.1469-8137.2006.01875.x

[CIT0028] Miki-Hiroshige H Hoek IHS Nakamura S 1987 Secretions from the pistil of *Lilium longiflorum* . American Journal of Botany 74, 1709–1715

[CIT0029] Muschietti J Eyal Y McCormick S 1998 Pollen tube localization implies a role in pollen-pistil interactions for the tomato receptor-like protein kinases LePRK1 and LePRK2. Plant Cell 10, 319–330 9501107 10.1105/tpc.10.3.319 PMC143994

[CIT0030] Nieuwland J Feron R Huisman BAH Fasolino A Hilbers CW Derksen J Mariani C 2005 Lipid transfer proteins enhance cell wall extension in tobacco. Plant Cell 17, 2009–2019 15937228 10.1105/tpc.105.032094 PMC1167548

[CIT0031] Oh IS Park AR Bae MS Kwon SJ Kim YS Lee JE Kang NY Lee S Cheong H Parka OK 2005 Secretome analysis reveals an *Arabidopsis* lipase involved in defense against *Alternaria brassicicola* . Plant Cell 17, 2832–2847 16126835 10.1105/tpc.105.034819 PMC1242276

[CIT0032] Ohmiya Y Samejima M Shiroishi M Amano Y Kanda T Sakai F Hayashi T 2000 Evidence that endo-1,4-β-glucanases act on cellulose in suspension-cultured poplar cells. The Plant Journal 24, 147–158 11069690 10.1046/j.1365-313x.2000.00860.x

[CIT0033] Panavas T Pikula A Reid PD Rubinstein B Walker EL 1999 Identification of senescence-associated genes from daylily petals. Plant Molecular Biology 40, 237–248 10412903 10.1023/a:1006146230602

[CIT0034] Park SY Jauh GY Mollet JCl Eckard KJ Nothnagel EA Walling LL Lord EM 2000 A lipid transfer-like protein is necessary for lily pollen tube adhesion in an in vitro stylar matrix. Plant Cell 12, 151–164 10634914 10.1105/tpc.12.1.151 PMC140221

[CIT0035] Parker JS Cavell AC Dolan L Roberts K Grierson CS 2000 Genetic interactions during root hair morphogenesis in *Arabidopsis* . Plant Cell 12, 1961–1974 11041890 10.1105/tpc.12.10.1961 PMC149133

[CIT0036] Petersen TN Brunak S von Heijne G Nielsen H 2011 SignalP 4.0: discriminating signal peptides from transmembrane regions. Nature Methods 8, 785–786 21959131 10.1038/nmeth.1701

[CIT0037] Ridley BL O’Neill MA Mohnen DA 2001 Pectins: structure, biosynthesis, and oligogalacturonide related signaling. Phytochemistry 57, 929–967 11423142 10.1016/s0031-9422(01)00113-3

[CIT0038] Röckel N Wolf S Kost B Rausch T Greiner S 2008 Elaborate spatial patterning of cell-wall PME and PMEI at the pollen tube tip involves PMEI endocytosis, and reflects the distribution of esterified and de-esterified pectins. The Plant Journal 53, 133–143 17971035 10.1111/j.1365-313X.2007.03325.x

[CIT0039] Roggen HPJR Stanley RG 1969 Cell-wall-hydrolysing enzymes in wall formation as measured by pollen-tube extension. Planta 84, 295–303 10.1007/BF00396421 24515494

[CIT0040] Ruepp A Zollner A Maier D 2004 The FunCat, a functional annotation scheme for systematic classification of proteins from whole genomes. Nucleic Acids Research 32, 5539–5545 15486203 10.1093/nar/gkh894 PMC524302

[CIT0041] Sanchez AM 2004 Pistil factors controlling pollination. Plant Cell 16, S98–S106 15010514 10.1105/tpc.017806 PMC2643392

[CIT0042] Sang YL Xu M Ma FF Chen H Xu XH Gao XQ Zhang XS 2012 Comparative proteomic analysis reveals similar and distinct features of proteins in dry and wet stigmas. Proteomics 12, 1983–1998 22623354 10.1002/pmic.201100407

[CIT0043] Shi H Kim Y Guo Y Stevenson B Zhu J-K 2003 The Arabidopsis SOS5 locus encodes a putative cell surface adhesion protein and is required for normal cell expansion. Plant Cell 15, 19–32 12509519 10.1105/tpc.007872 PMC143448

[CIT0044] Solomon M Belenghi B Delledonne M Menachem E Levine A 1999 The involvement of cysteine proteases and protease inhibitor genes in the regulation of programmed cell death in plants. Plant Cell 11, 431–444 10072402 10.1105/tpc.11.3.431 PMC144188

[CIT0045] Suárez C Castro AJ Rapoport HF Rodríguez-García MI 2012 Morphological, histological and ultrastructural changes in the olive pistil during flowering. Sexual Plant Reproduction 25, 133–146 22476326 10.1007/s00497-012-0186-3

[CIT0046] Takeda H Yoshikawa R Liu XZ Nakagawa N Li YQ Sakurai N 2004 Molecular cloning of two exo-beta-glucanases and their *in vivo* substrates in the cell walls of lily pollen tubes. Plant Cell Physiology 45, 436–444 15111718 10.1093/pcp/pch049

[CIT0047] Tang W Ezcurra I Muschietti J McCormick S 2002 A cysteine-rich extracellular protein, LAT52, interacts with the extracellular domain of the pollen receptor kinase LePRK2. Plant Cell 14, 2277–2287 12215520 10.1105/tpc.003103 PMC150770

[CIT0048] Tang W Kelley D Ezcurra I Cotter R McCormick S 2004 LeSTIG1, an extracellular binding partner for the pollen receptor kinases LePRK1 and LePRK2, promotes pollen tube growth *in vitro* . The Plant Journal 39, 343–353 15255864 10.1111/j.1365-313X.2004.02139.x

[CIT0049] The Gene Ontology Consortium 2010 http://www.geneontology.org

[CIT0050] Updegraff EP Zhao F Preuss D 2009 The extracellular lipase EXL4 is required for efficient hydration of *Arabidopsis* pollen. Sexual Plant Reproduction 22, 197–204 20033440 10.1007/s00497-009-0104-5

[CIT0051] Van der Hoorn RA 2008 Plant proteases: from phenotypes to molecular mechanisms. Annual Reviews in Plant Biology 59, 191–223 10.1146/annurev.arplant.59.032607.092835 18257708

[CIT0052] Van Hengel AJ Tadesse Z Immerzeel P Schols H van Kammen Ab de Vries SC 2001 *N*-Acetylglucosamine and glucosamine-containing arabinogalactan proteins control somatic embryogenesis. Plant Physiology 125, 1880–1890 11299367 10.1104/pp.125.4.1880 PMC88843

[CIT0053] Van Hengel AJ Van Kammen A De Vries SC 2002 A relationship between seed development, arabinogalactan-proteins (AGPs) and the AGP mediated promotion of somatic embryogenesis. Physiologia Plantarum 114, 637–644 11975739 10.1034/j.1399-3054.2002.1140418.x

[CIT0054] Vázquez-Lobo A Roujol D Zuñiga-Sánchez E Albenne C Piñero D Gamboa de Buen A Jamet E 2012 The highly conserved spermatophyte cell wall DUF642 protein family: phylogeny and first evidence of interaction with cell wall polysaccharides *in vitro* . Molecular and Phylogenetic Evolution 63, 510–520 10.1016/j.ympev.2012.02.001 22361214

[CIT0055] Verhoeven T Feron R Wolters-Arts M Edqvist J Gerats T Derksen J Mariani C 2005 STIG1 controls exudate secretion in the pistil of petunia and tobacco. Plant Physiology 138, 153–160 15821148 10.1104/pp.104.054809 PMC1104171

[CIT0056] Vieira M Pissarra J Veríssimo P Castanheira P Costa Y Pires E Faro C 2001 Molecular cloning and characterization of a cDNA encoding cardosin B, an aspartic proteinase accumulating extracellularly in the transmitting tissue of *Cynara cardunculus* L. Plant Molecular Biology 45, 529–539 11414612 10.1023/a:1010675015318

[CIT0057] Voigt J Frank R 2003 14-3-3 proteins are constituents of the insoluble glycoprotein framework of the chlamydomonas cell wall. Plant Cell 15, 1399–1413 12782732 10.1105/tpc.010611 PMC156375

[CIT0058] Wakelin AM Leung DWM 2009 β-1,3-Glucanase activity in the stigma of healthy petunia flowers. Biologia Plantarum 53, 69–74

[CIT0059] Wen F Van Etten HD Tsaprailis G Hawes MC 2007 Extracellular proteins in pea root tip and border cell exudates. Plant Physiology 143, 773–783 17142479 10.1104/pp.106.091637 PMC1803736

[CIT0060] White RG Badelt K Overall RL Vesk M 1994 Actin associated with plasmodesmata. Protoplasma 180, 169–184

[CIT0061] Wilkins KA James Bancroft Bosch M Ings J Smirnoff N Franklin-Tong VE 2011 Reactive oxygen species and nitric oxide mediate actin reorganization and programmed cell death in the self-incompatibility response of *Papaver* . Plant Physiology 156, 404–416 21386034 10.1104/pp.110.167510 PMC3091060

[CIT0062] Wolters-Arts M Van Der Weerd L Van Aelst AC Van Der Weerd J Van As H Mariani C 2002 Water-conducting properties of lipids during pollen hydration. Plant Cell, & Environment 25, 513–519

[CIT0063] Woodward JR Craik D Dell A Khoo K-H Munro SLA Clarke AE Bacic A 1992 Structural analysis of the N-linked glycan chains from a stylar glycoprotein associated with expression of self-incompatibility in *Nicotiana alata* . Glycobiology 2, 241–250 1498421 10.1093/glycob/2.3.241

[CIT0064] Wu HM Cheung AY 2000 Programmed cell death in plant reproduction. Plant Molecular Biology 44, 267–281 11199388 10.1023/a:1026536324081

[CIT0065] Yokota E Ohmori T Muto S Shimmen T 2004 21-kDa polypeptide, a low-molecular-weight cyclophilin, is released from pollen of higher plants into the extracellular medium *in vitro* . Planta 218, 1008–1018 14745555 10.1007/s00425-003-1177-2

[CIT0066] Zhu JK Damsz B Kononowicz AK Bressan RA Hasegawa PM 1994 A higher plant extracellular vitronectin-like adhesion protein is related to the translational elongation factor-1α. Plant Cell 6, 393–404 7514059 10.1105/tpc.6.3.393 PMC160442

