# Supplementary material for: Proteomics profiling reveals novel proteins and functions of the plant stigma exudate
Source: J Exp Bot. 2013 Oct 22;64(18):5695–705. doi: 10.1093/jxb/ert345 (PMC3871823; doi:10.1093/jxb/ert345)
Supplement: Supplementary Data [file supp_64_18_5695__index.html]

Proteomics profiling reveals novel proteins and functions of the plant stigma exudate — Proteomics profiling reveals novel proteins and functions of the plant stigma exudate — Supplementary Data 

# Proteomics profiling reveals novel proteins and functions of the plant stigma exudate

## Supplementary Data

Data files

**Files in this Data Supplement:**

- Supplementary Data - Supplementary Data
- Supplementary Data - Supplementary Data
- Supplementary Data - Supplementary Data
