# Supplementary material for: Proteomics profiling reveals novel proteins and functions of the plant stigma exudate
Source: J Exp Bot. 2013 Oct 22;64(18):5695–705. doi: 10.1093/jxb/ert345 (PMC3871823; doi:10.1093/jxb/ert345)
Supplement: Supplementary Data [file supp_ert345_jexbot106898_file003.pdf]

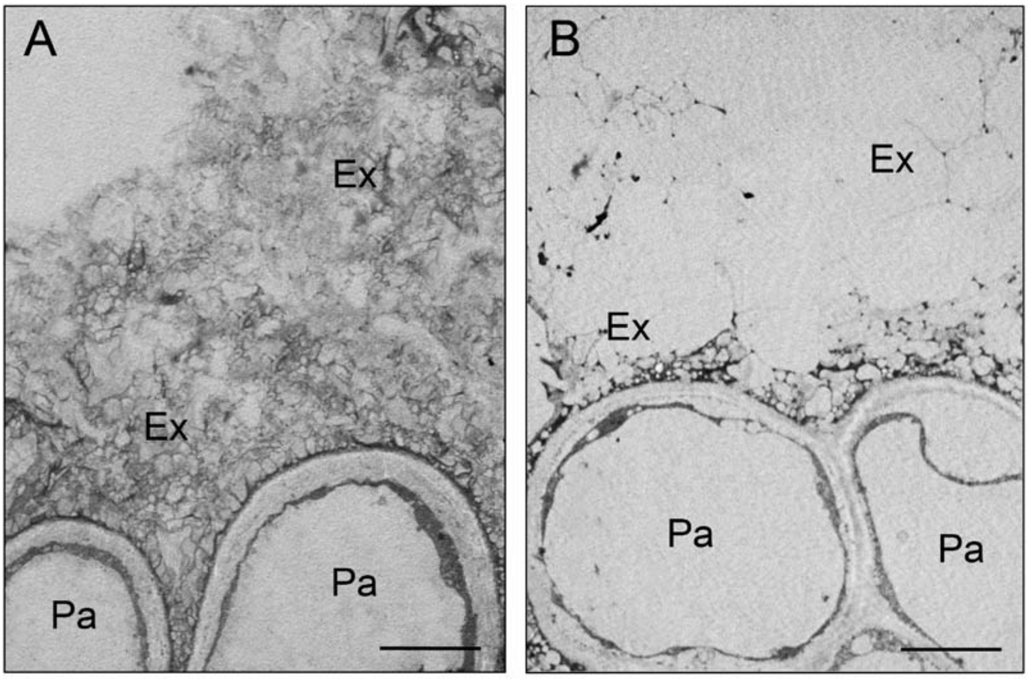

Supplementary figure S1. (A) Transmission electron microscopy (TEM) photomicrograph of an olive stigma before pollination showing papillae ultrastructure and the presence of a copious exudate covering its surface. (B) TEM photomicrograph as above showing intact papillae after exudate collection by brushing.
